# Association of *p53* rs1042522, *MDM2* rs2279744, and *p21* rs1801270 polymorphisms with retinoblastoma risk and invasion in a Chinese population

**DOI:** 10.1038/srep13300

**Published:** 2015-08-20

**Authors:** Rongxin Chen, Shu Liu, Huijing Ye, Jiali Li, Yi Du, Lingyan Chen, Xiaoman Liu, Yungang Ding, Qian Li, Yuxiang Mao, Siming Ai, Ping Zhang, Wenfang Ma, Huasheng Yang

**Affiliations:** 1 State Key Laboratory of Ophthalmology, Zhongshan Ophthalmic Center, Sun Yat-sen University, Guangzhou 510060, China; 2 Sun Yat-sen University Cancer Center, State Key Laboratory of Oncology in South China, Collaborative Innovation Center for Cancer Medicine, Guangzhou 510060, China; 3 Institute of Clinical Pharmacology, School of Pharmaceutical Sciences, Sun Yat-sen University, Guangzhou 510060, China; 4 Department of Ophthalmology, The First Affiliated Hospital of Guangxi Medical University, Nanning, Guangxi 530021, China; 5 Divisions of Genetics and Molecular Medicine, King’s College London, Guy’s Hospital, London SE1 9RT, UK

## Abstract

Single nucleotide polymorphisms (SNPs) of *p53* rs1042522, *MDM2* rs2279744 and *p21* rs1801270, all in the p53 pathway, which plays a crucial role in DNA damage and genomic instability, were reported to be associated with cancer risk and pathologic characteristics. This case-control study was designed to analyse the association between these SNPs and retinoblastoma (RB) in a Chinese Han population. These SNPs in 168 RB patients and 185 adult controls were genotyped using genomic DNA from venous blood. No significant difference was observed in allele or genotypic frequencies of these SNPs between Chinese RB patients and controls (all *P *> 0.05). However, the rs1042522 GC genotype showed a protective effect against RB invasion, as demonstrated by event-free survival (HR = 0.53, *P* = 0.007 for GC versus GG/CC). This effect was significant for patients with a lag time >1 month and no pre-enucleation treatment (*P* = 0.007 and *P* = 0.010, respectively), indicating an interaction between *p53* rs1042522 and clinical characteristics, including lag time and pre-enucleation treatment status. Thus, the rs1042522 SNP may be associated with RB invasion in the Han Chinese population; however, further large and functional studies are needed to assess the validity of this association.

Retinoblastoma (RB), a rare eye tumour observed in paediatric patients, is the most common intraocular malignancy among children worldwide^1^. Its global annual incidence is one case per 15,000–20,000 newborns—an estimated 9,000 new cases every year^2^. Both germinal/heritable (40%) and non-germinal/non-heritable RB (60%) originate in the primordial retinal cells and are predominantly initiated by biallelic inactivation of the *RB1* gene^1^. The deletion of Rb family members leads to the compensatory up-regulation of the cyclin-dependent kinase inhibitor^3^ p21, which is the primary protein upregulated by p53 in response to DNA damage^4^. The p53 pathway, which is the master control system of the cell cycle, genome stability, and cell apoptosis, is controlled by a negative feedback loop in which p53 transcriptionally activates MDM2, which in turn functions as a negative regulator by promoting the proteolytic degradation of p53^5^. In addition to the loss of function of the *RB1* gene, several other genes have been shown to be dysregulated in RB cells^6^,^7^,^8^ through single nucleotide polymorphisms (SNPs) in *p53* pathway genes and may influence RB cancer risk among other ethnic populations^9^,^10^,^11^,^12^,^13^, indicating that SNPs in the *p53* pathway genes and *RB1* gene mutations may coincide in RB.

The Arg to Pro change in codon 72 (also known as p.Arg72Pro, rs1042522 G > C) is the most frequently studied functional SNP in *p53*. Compared with the G allele, the C allele of rs1042522 in *p53* reduces proapoptotic activity and ultimately mediates the attenuation of the p53 pathway in concert with its induction of the *MDM2* gene and other p53 targets^14^. An explanation of this diminished proapoptotic activity for the C allele is suggested to be a high affinity binding of the inhibitor of the apoptosis-stimulating protein of the p53 family^15^. Furthermore, the rs1042522 SNP modulates susceptibility to several human cancers, including hereditary RB in an Italian population^10^. In addition, the G allele has been associated with increased tumour stage^16^,^17^,^18^ and higher invasive or metastatic risk of cancer^19^,^20^. For the *MDM2* gene, the G allele of a naturally occurring SNP at position 309 in the *MDM2* promoter (also known as c.14 + 309T > G, rs2279744 T > G) has been shown to produce a higher-affinity DNA-binding site for Sp1, which leads to increased MDM2 mRNA and protein and thus increases the degradation of p53 and hinders p53-induced apoptosis^21^. Increased MDM2 expression is related to elevated cancer risk in sporadic and hereditary malignancies and to an increased likelihood of distant metastases^22^, and rs2279744 in the *MDM2* gene is also associated with RB development^9^,^10^,^11^. Overall, these data indicate a risk-modifying effect of *p53* and *MDM2* SNPs on cancer^23^,^24^ and an association of these SNPs with pathological tumour characteristics^25^,^26^.

p21 is the primary protein that is upregulated by activated p53 in response to DNA damage^4^; conversely, p21 acts as a negative regulator of p53 stability^27^. Moreover, MDM2 negatively regulates p21 protein stability via proteasome-mediated degradation^28^. Most studies have focused on the *p21* SNP in codon 31 (also known as p.Ser31Arg, rs1801270 C > A), which results in a non-synonymous serine-to-arginine substitution in the DNA-binding zinc finger motif of the protein^29^ and can lead to the failure of p21 to accumulate upon DNA damage, despite a normal p53 protein response^30^. Thus, this *p21* SNP may affect the expression and activity of p21, thereby disturbing the p53 pathway activity and playing a role in the susceptibility to cancers^31^ including RB^13^.

Additionally, the p53 pathway targets pRB for degradation and controls the cell cycle and apoptosis in retinal cone precursor cells, from which the RB cell lineage originates^32^. Thus, the rs1042522, rs2279744, and rs1801270 SNPs in *p53* pathway genes may affect the fate of RB. Previous studies have highlighted the contribution of the common functional SNPs rs1042522, rs2279744, and rs1801270 to RB cancer risk in non-Chinese populations^9^,^10^,^11^,^12^,^13^; however, to our knowledge, the association between these SNPs and RB invasion has not been reported. In the current study, for the first time, we genotyped these SNPs (rs1042522, rs2279744, and rs1801270) and evaluated their association with RB cancer risk and tumour invasion in a Chinese population.

## Results

### Study-population

This study was conducted on 168 RB patients and 185 cancer-free adult controls. The mean age of all patients at presentation was 20.0 ± 16.6 months, with a range from 1.0 to 118 months. No significant difference in gender was detected between the RB patients and the controls (*P *= 0.081). The clinical characteristics of the RB patients are summarised in Table 1. According to the International Classification of Retinoblastoma (ICRB), in the 168 patients, 131 eyes from 131 patients were enucleated, including 67 eyes (51.1%) in hazard group D and 64 eyes (48.9%) in hazard group E. Among enucleated patients, the percentage who received pre-enucleation treatment was higher in bilateral RB (78.9%) than in unilateral RB (5.4%) (*P* = 0.000), and a significantly negative correlation was observed between the age at presentation and lag time or pre-enucleation treatment (*r *= −0.21, *P* = 0.018; *r *= −0.25, *P* = 0.004, respectively). Histopathology reports were retrospectively staged for pTNM-staging characteristics, and re-examination of the histopathological findings by a second reviewer (R.C.) showed 100% concurrence regarding the grade of RB invasion. Of the 131 enucleated eyes, 37 (28.2%), 49 (37.4%), and 45 (34.4%) enucleated eyes showed no, moderate, and high invasion, respectively.

### rs1042522, rs2279744, and rs1801270 SNPs and RB cancer risk

**Figure 1:**
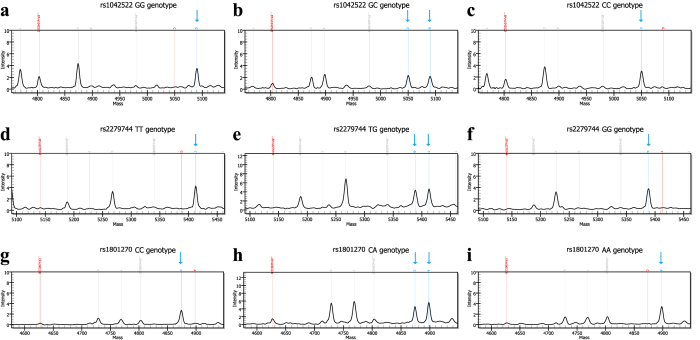
Typical raw data obtained using the Sequenom MassARRAY system for single nucleotide polymorphisms of (**a**–**c**) *p53* rs1042522 G/C, (**d**–**f**) *MDM2* rs2279744 T/G and (**g**–**i**) *p21* rs1801270 C/A. The blue arrows above the wave peak indicate the resulting genotypes.

The *p53* rs1042522, *MDM2* rs2279744, and *p21* rs1801270 SNPs were clearly distinguished by matrix-assisted laser desorption/ionisation time-of-flight (MALDI-TOF) mass spectrometry (MS), and the success rates of the genotype calls for these SNPs were 100%, 99.7%, and 100%, respectively. Representative spectral peak diagrams are depicted in Fig. 1. In the control group, the genotype distributions of the evaluated SNPs were consistent with Hardy-Weinberg equilibrium (HWE) (all *P* > 0.05, Table 2). As shown in Table 2, neither the minor allele frequencies (MAFs) nor the genotype frequencies of the three selected SNPs were significantly different between RB and control groups (all *P* > 0.05).

### rs1042522, rs2279744, and rs1801270 SNPs and RB invasion

Briefly, the distributions of the analysed genotypes were not significantly associated with any clinical characteristics of the patients (see Supplementary Table S1), except for the distribution of rs1042522 genotypes in laterality and pre-enucleation treatment status (*P* = 0.001 and *P* = 0.013, respectively) and of rs2279744 genotypes in lag time (*P* = 0.007); we adjusted for these factors in the Cox regression model analysis. For the patients with enucleated eyes, the analyses revealed almost identical associations of the selected SNPs with moderate and high invasion (see Supplementary Table S2). Therefore, the moderate and high invasion groups were combined into one group.

**Figure 2:**
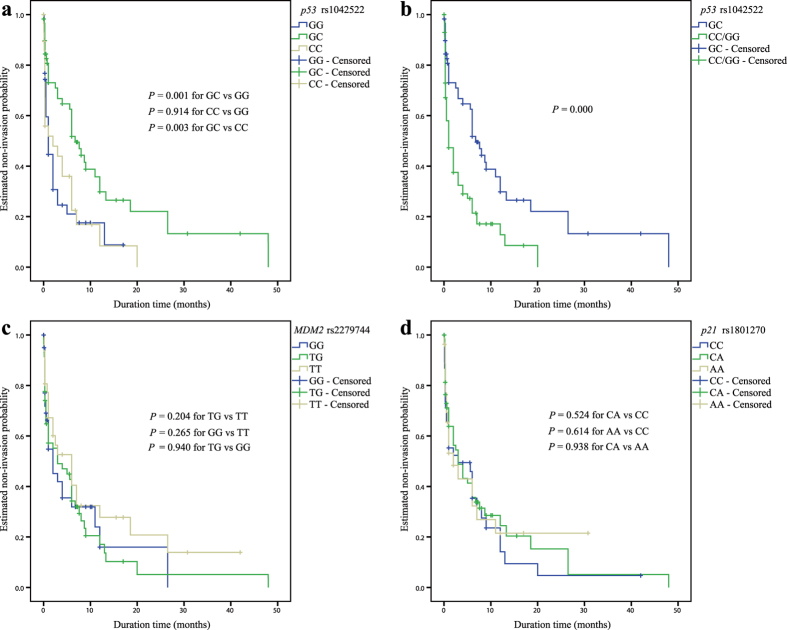
Kaplan-Meier analysis of the association of the selected SNPs of *p53* pathway genes with event-free survival (EFS) for RB invasion among enucleated patients. (**a**) Cumulative EFS curve of the enucleated patients for the three genotypes of *p53* rs1042522 (*P* = 0.001 for GC versus GG, *P* = 0.914 for CC versus GG, and *P* = 0.003 for GC versus CC); (**b**) Cumulative EFS curve of enucleated patients according to the over-dominant genetic model of *p53* rs1042522 (*P* = 0.000 for GC versus GG/CC). (**c**) Cumulative EFS curve of enucleated patients according to the three genotypes of *MDM2* rs2279744 (*P* = 0.204 for TG versus TT, *P* = 0.265 for GG versus TT, and *P* = 0.940 for TG versus GG); (**d**) Cumulative EFS curve of enucleated patients according to the three genotypes of *p21* rs1801270 (*P* = 0.524 for CA versus CC, *P* = 0.614 for AA versus CC, and *P* = 0.938 for CA versus AA).

As shown in Fig. 2, a significant association was observed only for the rs1042522 SNP. Relative to the combined homozygous genotypes (GG and CC) in an over-dominant genetic model, the heterozygous GC genotype of *p53* rs1042522 was significantly associated with event-free survival (EFS) for RB invasion (*P* = 0.000, Fig. 2a-b). For the genotypic association analysis of *p21* rs1801270 and *MDM2* rs2279744, no significant association with EFS was observed (Fig. 2c-d).

The results of the multivariate analysis of EFS using the Cox proportional hazards model are presented in Table 3. Only the carriers of the *p53* rs1042522 GC genotype exhibited a significantly decreased risk of RB invasion (HR = 0.53; 95% CI = 0.33–0.84; *P* = 0.007) after adjustment for age (≤12 or >12 months), gender (male or female), laterality (unilateral or bilateral), clinical predictors (whether presented), ICRB (group D or E), lag time (≤1 or >1 month), and pre-enucleation treatment (administered or not). Furthermore, a lag time greater than 1 month and no pre-enucleation treatment were also associated with a reduced risk of RB invasion based on multivariate analysis (HR = 0.10, 95% CI = 0.05–0.24, *P* = 0.000, and HR = 0.02, 95% CI = 0.05–0.24, *P* = 0.000, respectively); a significant association was observed between age older than 12 months or bilateral eyes and the risk of RB invasion in the univariate analyses, whereas no association was observed in the multivariate analyses (HR = 1.13, 95% CI = 0.68–1.88, *P *= 0.635, and HR = 1.38, 95% CI = 0.623–3.11, *P* = 0.431, respectively). However, no significant association was detected between the genotypes of the other two SNPs and the risk of RB invasion.

Moreover, a subgroup analysis of the association of *p53* rs1042522 with RB invasion risk was performed according to lag time and pre-enucleation treatment status. As shown in Table 4, in only the separate analyses of subgroups of lag time greater than 1 month and no prior treatment, the patients exhibited a significantly increased EFS duration (*P* = 0.008, and *P *= 0.004, respectively) and reduced invasion risk (HR = 0.31, *P *= 0.007, and HR = 0.50, *P* = 0.010, respectively) for the GC carriers relative to the homozygous carriers. However, no significant association between the *MDM2* or *p21* genotypes and RB invasion was observed according to the subgroup analyses of lag time or pre-enucleation treatment (see Supplementary Table S3).

## Discussion

This study reports for the first time that the SNPs rs1042522, rs2279744, and rs1801270 in *p53* pathway genes are not correlated with RB cancer risk, but rs1042522 is associated with RB invasion in a Chinese Han population. RB was classified according to the extent of tumour invasion to determine the association of RB invasion with these SNPs. The heterozygous GC genotype of *p53* rs1042522 was negatively associated with RB invasion among patients with a lag time greater than 1 month and among those who had not undergone pre-enucleation treatments; however, no associations were observed between *MDM2* rs2279744 or *p21* rs1801270 and invasion.

Several studies have proposed a role for the *p53* rs1042522 SNP as a risk factor for cancer due to its influence on cellular processes via p53-induced apoptosis^33^. Although the C allele frequency in Chinese RB (0.443) was relatively higher than that found in an Italian population (0.32) described in a previous report^10^, there was no association between the MAF of rs1042522 and RB cancer risk. This finding may be the result of a relatively high frequency of the C allele in the controls (0.438), which was similar to the C allele frequency of 0.489 in the Chinese population (HapMap-CHB database, http://hapmap.ncbi.nlm.nih.gov). Additionally, no association between RB cancer risk and the rs1042522 genotypes was found in this study. This finding was not in agreement with a previous study, which found an association between the *p53* CC genotype and hereditary RB among 111 patients in an Italian population^10^. At present, there is no mechanism to support any proposed role for the rs1042522 SNP as a risk factor for RB, and this lack of evidence may be due to conflicting results from different studies, which may also be attributed to the diverse distributions of SNPs in different races.

In addition, the mean age at presentation to our centre was 20.0 months, which was approximately the same as that in other Asian populations^34^,^35^,^36^. For the enucleated patients, those older than 12 months showed an increased risk of RB invasion based on univariate analysis (*P *= 0.001); this result was in accord with a previous study showing that older age is strongly associated with a high risk of histopathological findings^37^, whereas age was correlated with pre-enucleation treatment status and lag time in our study. Meanwhile, our data are consistent with a previous finding that a relatively higher percentage of RB patients initially presented with unilateral RB^34^,^36^. The enucleated patients with bilateral RB exhibited a reduced risk of RB invasion in the univariate analysis (*P* = 0.000), whereas the patients with bilateral RB tended to require pre-enucleation treatment in our study. An explanation of these findings is that chemotherapy before the enucleation of eyes containing RB diminishes the pathologic characteristics of extraocular extension^38^, and the distribution of *p53* rs1042522 genotypes was correlated with RB laterality, pre-enucleation treatment status, and lag time. For these reasons, the RB invasion risk was evaluated using multivariate analysis. After adjustment for clinical characteristics, the heterozygous genotype of *p53* rs1042522 remained significantly associated with decreased RB invasion (*P* = 0.007).

Furthermore, subgroup analyses supported the association between the *p53* rs1042522 GC genotype and RB invasion within the subgroup of patients with a lag time greater than 1 month (*P *= 0.007) or without prior treatment (*P* = 0.010). Therefore, an interaction between *p53* rs1042522 genotypes and clinical characteristics (lag time and pre-enucleation treatment) contributed to RB invasion. This finding may be related to a decreased risk of future RB invasion because this result was observed in patients with a less invasive phenotype. These results may represent a “heterozygote advantage” situation in which the heterozygotes exhibit favourable survival characteristics^39^. Similar results have been observed in sickle cell anaemia^40^, HPV status in women from South Brazil,^41^ and breast cancer in an Iranian Azeri population^42^. Therefore, our findings may be useful in the near future for the promotion of prevention strategies guided by the patient’s genotype because the *p53* rs1042522 GC genotype was associated with reduced RB invasion (over-dominant model). However, contrary to our finding, the GC genotype of rs1042522 showed a “heterozygote disadvantage” in which the heterozygote displayed an increased risk of metastasis in breast-invasive ductal carcinoma, based on a study conducted in northeast Brazil^43^. Although two wild-type alleles of rs1042522 have different potentials in binding components of the transcriptional machinery, activating transcription, inducing apoptosis, and repressing cancer cells, recent studies demonstrate that rs1042522 could also be associated with cancer through an epigenetic mechanism, including RNAi suppression^44^ and DNA methylation^45^. Thus, the exact biological and genetic mechanisms underlying such associations are uncertain, and further studies are necessary to confirm the association of the rs1042522 GC genotype with RB invasion.

Although the *p53* gene is not mutated in primary RB^46^, the T-to-G variant of *MDM2* has been shown to result in increased transcription, which plays a crucial role in the p53 pathway and in accelerating tumour formation^21^,^47^,^48^,^49^,^50^. The lack of an association of the *MDM2* rs2279744 SNP contrasts with reports showing that the G allele appears to protect against RB development^9^,^11^. Moreover, our experiment did not show any association between the *MDM2* rs2279744 SNP and RB invasion or the patients’ clinical characteristics. Thus, our findings appear to be plausible, considering a previous report showing that this association is only observed in *p53*-mutated cancers^51^.

Contrary to several molecular epidemiological studies that have reported the effects of the *p21* rs1801270 SNP on cancer risk, no increased risk of developing RB was found in the studied Chinese population (*P* = 0.712). This finding contradicted the increased RB risk shown in Brazilian carriers of the CA genotype^13^. Although the *p21* rs1801270 A allele results in a 38% reduction of p21 mRNA expression^52^, we found no association between the rs1801270 SNP and RB invasion based on both univariate and multivariate analyses (adjusted or not adjusted for the clinical characteristics of the patients) of the overall data. These results may partly account for the variation in the data regarding the MAF of rs1801270 in the population, ranging from 4% in a Swedish population to 50% in a Chinese population^53^. Compared to the frequency of the A allele (21%) in RB patients, which was reported by Carvalho *et al.*, a relatively high A allele frequency (45.5%) was found in our study.

In conclusion, we have demonstrated that the *p53* rs1042522 GC genotype, but not the *MDM2* rs2279744 or *p21* rs1801270 genotypes, is associated with a reduced risk of RB invasion in patients with a lag time greater than 1 month or in patients who received no prior treatment before enucleation, suggesting an interaction of rs1042522 with lag time and pre-enucleation treatment status in RB invasion. Considering how these SNPs interact with germline mutations in the *RB1* gene, the present findings—if validated in other populations—could provide novel therapeutic implications.

## Methods

### Ethics statement

This study was approved by the medical ethics committee of Zhongshan Ophthalmic Center in Guangzhou (No. 2013PRLL019). Additionally, informed consent was obtained from all individuals after a thorough explanation of the procedure and its risk in accordance with the principles of the Declaration of Helsinki.

### Study population

A total of 168 RB patients and 185 cancer-free controls were included in the present study. In brief, the patients were consecutively recruited from Zhongshan Ophthalmic Center of Sun Yat-sen University, Guangzhou, China, between February 2013 and October 2014. Unrelated controls of similar gender and ethnicity were recruited from healthy adults who visited the clinic for a general health check-up and had no individual history of cancer. Additionally, all subjects included in this study were Han Chinese. Subsequently, a peripheral blood sample was obtained from each recruited individual for genomic DNA extraction, and the clinical information about the RB patients (age at presentation, gender, family history of RB, lag time between first symptom and treatment onset, laterality, clinical predictors at presentation, ICRB classification^54^, therapeutic regimen (including chemotherapy, focal treatment and enucleation), histopathology reports, and duration between first symptom and enucleation onset) was collected from their medical records. The included clinical predictors were glaucoma, pseudohypopyon, hyphaema, staphyloma, and neovascularisation of the iris^37^,^55^.

### Assessment of RB invasion

The extent of tumour invasion into the anterior chamber, the iris, the ciliary body, the choroid, the sclera, the extra-scleral tissue, the optic nerve preceding or beyond the lamina, and the optic nerve section margin were recorded based on written pathology reports and were confirmed via a retrospective re-review of haematoxylin and eosin-stained slides. For some analyses, the enucleation group was further subdivided into the following three groups based on the AJCC pTNM stage of RB invasion: no (pT1), moderate (pT2), or high (pT3, pT4) tumour invasion^56^ (Table 5).

### Laboratory methods

#### Total DNA extraction

Total genomic DNA was extracted from 100 μL of EDTA-Na_2_ anticoagulated peripheral blood samples from each individual according to a previously described protocol^57^. The extracted DNA was eluted in 40 μL of TE buffer and stored at −20 °C in a dedicated area that was only used for PCR. The absorbance ratio of 260 nm to 280 nm was used to assess the purity of the isolated DNA using a Multiskan GO Microplate Spectrophotometer (Thermo Fisher Scientific, Inc., Waltham, MA, USA), and an absorbance ratio of ~1.8 was generally accepted as “pure”.

*Genotyping.* Based on the Sequenom Mass-ARRAY iPLEX platform, the SNP alleles were detected by allele-specific primer extension products using MALDI-TOF MS (Sequenom Inc., San Diego, California, USA). A multiplexed SNP assay was designed to test up to 3 SNPs using the “MySequenom” online design tools (https://www.mysequenom.com/Tools). According to the designated specifications, polymerase chain reaction (PCR) amplification primers and single-base extension (SBE) oligonucleotides for each SNP assay were synthesised and purified by Sangon Biotech Co., Ltd (Shanghai, China). PCR primers within the same multiplex were mixed at a final concentration of 1 μM each, and the final concentrations of the SBE oligonucleotides were optimised according to the recommendations.

The genotyping reactions were performed on batches of 96-well plates according to the manufacturer’s instructions (http://www.sequenom.com). For PCR amplification, 10 ng of genomic DNA, dNTPs, primer mix, and HotStart Taq polymerase (Qiagen Inc., Valencia, CA) were added to each well, and the DNA was amplified via multiplex PCR. Unincorporated dNTPs present in the PCR product were neutralised using shrimp alkaline phosphatase (SAP), which was supplied with the iPLEX Gold reagent kit (Sequenom Inc.). After the SAP reaction, the SBE reactions were performed by adding extension primers, DNA polymerase, and di-deoxynucleotide triphosphates (Sequenom Inc.) to each well. Subsequently, clean resin (Sequenom Inc.) was added to the mixture to remove extraneous salts that could interfere with MALDI-TOF analysis. The primer extension products were cleaned, and aliquots of each sample were dispensed onto a 96-pad SpectroCHIP (Sequenom Inc.). Ultimately, genotype calling was performed in real time using MassARRAY RT software (version 3.0.0.4, Sequenom Inc.), and the results were analysed using MassARRAY Typer software (version 4.0, Sequenom Inc.). Duplicate samples and negative controls were included to verify the genotyping quality.

### Statistical analysis

HWE of the genotype frequencies was tested using a goodness-of-fit Chi-square test with one degree of freedom among the controls. Comparisons of the SNPs between groups were analysed using Pearson’s Chi-square tests. Considering the major allele as the reference, logistic regression analysis was used to calculate the odds ratios (OR) and their corresponding 95% confidence intervals (CIs), which assessed the relative RB cancer risk conferred by each of the genotypes and alleles. In the univariate analysis, each genotype was independently analysed for a correlation with the duration of EFS for RB invasion. The Kaplan-Meier method was performed to estimate survival curves, and the log-rank test was used to compare the EFS duration between the subgroups of RB patients. In the multivariate analysis, the association between the genetic models of the selected SNPs and RB invasion risk was estimated by computing the hazard ratio (HR) and the respective 95% CI using a Cox proportional hazards model, which was adjusted for potential risk factors that were significantly or marginally associated with invasion risk in the univariate analysis. If a *P* value was less than 0.05, the corresponding factor was included as a covariate in the Cox regression analysis. In the association analysis, all factors displaying *P* < 0.05 were subjected to subgroup analysis. All statistical tests were two-sided and were performed using SPSS version 20.0 for Mac OS (IBM Corporation, Armonk, New York, USA). The *P* value was set at a significance level of 0.05. For all multivariate analyses, the Bonferroni correction was applied.

## Additional Information

**How to cite this article**: Chen, R. *et al.* Association of *p53* rs1042522, *MDM2* rs2279744, and *p21* rs1801270 polymorphisms with retinoblastoma risk and invasion in a Chinese population. *Sci. Rep.* **5**, 13300; doi: 10.1038/srep13300 (2015).

## Supplementary Material

Supplementary Information

## Figures and Tables

**Table 1:** Clinical characteristics of the patients with retinoblastoma.

Variables	Total patients N = 168	Patients who received enucleation N = 131
Age at diagnosis (months)		
≤12 months	64 (38.1)	42 (32.1)
>12 months	104 (61.9)	89 (67.9)
Gender		
Male	102 (60.7)	80 (61.1)
Female	66 (39.3)	51 (38.9)
Family history of RB		
Absent	163 (97.0)	127 (96.9)
Present	5 (3.0)	4 (3.1)
Lag time (months)		
Range (median)	0.0–48.0 (1.0)	0.0–48.0 (1.0)
Mean ± SD	2.8 ± 5.8	3.0 ± 6.3
Laterality		
Unilateral	97 (57.7)	92 (70.2)
Bilateral	71 (42.3)	39 (29.8)
Clinical predictors at presentation (eyes)		
Absent	171 (71.5)	78 (59.5)
Present	68 (28.5)	53 (40.5)
Pre-enucleation treatment		
No prior treatment		96 (73.3)
Systemic chemotherapy and/or focal treatment^a^		35 (26.7)
Tumour invasion		
No invasion		37 (28.2)
Moderate invasion		49 (37.4)
High invasion		45 (34.4)

The results are shown as the frequencies and percentages unless otherwise indicated.

^a^Focal treatment included periocular chemotherapy, laser photocoagulation, cryotherapy, or intra-arterial chemotherapy.

**Table 2:** Genotypic and allele frequencies of the selected SNPs in *p53* pathway genes among patients and controls and the associations of these SNPs with the risk of retinoblastoma.

SNPs	Patients (n = 168)		Controls (n = 185)		OR (95% CI)	*P*^a^	HWE
	n	%	n	%			*p*a,^b^
*p53* rs1042522							
GG	53	31.5	52	28.1	1.00 (reference)		0.053
GC	81	48.2	104	56.2	0.76 (0.47-1.24)	0.272	
CC	34	20.2	29	15.7	1.15 (0.62-2.15)	0.661	
C allele		44.3		43.8	1.02 (0.76-1.38)	0.881	
*MDM2* rs2279744							
TT	34	20.2	36	19.6	1.00 (reference)		0.715
TG	75	44.6	88	47.8	0.90 (0.52-1.58)	0.720	
GG	59	35.1	60	32.6	1.04 (0.58-1.88)	0.893	
T allele		42.6		43.5	0.96 (0.71-1.30)	0.806	
*p21* rs1801270							
CC	51	30.4	46	24.9	1.00 (reference)		0.712
CA	81	48.2	95	51.4	0.77 (0.47-1.26)	0.300	
AA	36	21.4	44	23.8	0.74 (0.41-1.34)	0.316	
A allele		45.5		49.5	0.85 (0.64-1.15)	0.297	

Abbreviations: SNPs, single nucleotide polymorphisms; OR, odds ratio; CI, confidence interval; HWE, Hardy-Weinberg equilibrium.

^a^*P*-value obtained from the Chi-squared test.

^b^*P*-value obtained from Hardy-Weinberg equilibrium of the control group.

**Table 3:** Analyses of event-free survival for retinoblastoma invasion using a Cox proportional hazards model.

Variable	No. of cases	No. of events (%)	Crude HR (95% CI)	*P*	Adjusted HR (95% CI)^a^	*P*^a^
Age at presentation						
≤12 months	42	24 (57.1)	1.00 (reference)		1.00 (reference)	
>12 months	89	70 (78.7)	2.36 (1.45-3.83)	0.001	1.13 (0.68-1.88)	0.635
Lag time						
≤1 month	83	58 (69.9)	1.00 (reference)		1.00 (reference)	
>1 month	45	34 (75.6)	0.47 (0.31-0.74)	0.001	0.10 (0.05-0.24)	0.000
Laterality						
Unilateral	93	73 (78.5)	1.00 (reference)		1.00 (reference)	
Bilateral	38	21 (55.3)	0.30 (0.18-0.50)	0.000	1.38 (0.62-3.11)	0.431
Pre-enucleation treatment						
No prior treatment	96	77 (80.2)	1.00 (reference)		1.00 (reference)	
Systemic chemotherapy and/or focal treatment	35	17 (48.6)	0.10 (0.05-0.20)	0.000	0.02 (0.004-0.05)	0.000
*p53* rs1042522						
GG/CC	72	56 (77.8)	1.00 (reference)		1.00 (reference)	
GC	59	38 (64.4)	0.46 (0.30-0.70)	0.000	0.53 (0.33-0.84)	0.007
*MDM2* rs2279744						
TT	31	22 (71.0)	1.00 (reference)		1.00 (reference)	
TG	58	45 (77.6)	1.40 (0.83-2.35)	0.204	1.04 (0.60-1.79)	0.888
GG	42	27 (64.3)	1.37 (0.78-2.43)	0.277	0.91 (0.50-1.65)	0.755
*p21* rs1801270						
CC	39	30 (76.9)	1.00 (reference)		1.00 (reference)	
CA	65	46 (70.8)	0.87 (0.55-1.38)	0.560	1.20 (0.73, 1.95)	0.471
AA	27	18 (66.7)	0.88 (0.49-1.57)	0.654	0.82 (0.45-1.51)	0.522

Abbreviations: CI, confidence interval; HR, hazard ratio.

^a^Adjusted for age, gender, laterality, clinical predictors, ICRB, lag time, and pre-enucleation treatment.

**Table 4:** Subgroup analyses of the association between the *p53* rs1042522 SNP and event-free survival for retinoblastoma invasion.

Genotype	Subgroup	Log-rank analysis^a^		Cox regression model^b^	
		Time to RB invasion (median ± SE, months)	*P*	HR (95% CI)	*P*
GG/CC	Lag time ≤1 month	0.5 ± 0.1		1.00 (reference)	
GC	Lag time ≤1 month	5.6 ± 2.4	0.007	0.64 (0.35-1.15)	0.134
GG/CC	Lag time >1 month	5.0 ± 1.0		1.00 (reference)	
GC	Lag time >1 month	8.0 ± 3.5	0.008	0.31 (0.13-0.73)	0.007
GG/CC	No prior treatment	0.5 ± 0.2		1.00 (reference)	
GC	No prior treatment	3.0 ± 0.8	0.004	0.50 (0.30, 0.85)	0.010
GG/CC	Pre-enucleation treatment	13.0 ± 1.0		1.00 (reference)	
GC	Pre-enucleation treatment	18.5 ± 6.0	0.587	0.25 (0.05, 1.14)	0.072

Abbreviations: SNP, single nucleotide polymorphism; SE, standard error.

^a^Crude analysis.

^b^The HRs and *P* values were adjusted for age, gender, laterality, clinical predictors, ICRB, lag time, and pre-enucleation treatment.

**Table 5:** The tumour invasion grade according to the T classification of pTNM.

Invasion grade	Definition
No invasion	
pTX	Primary tumour cannot be assessed
pT0	No evidence of primary tumour
pT1	Tumour confined to the eye, with no optic nerve or choroid invasion
Moderate invasion	
pT2a	Tumour superficially invades the optic nerve head but does not extend beyond the lamina cribrosa or exhibits focal choroidal invasion
pT2b	Tumour superficially invades the optic nerve head but does not extend beyond the lamina cribrosa and exhibits focal choroidal invasion
High invasion	
pT3a	Tumour invades the optic nerve beyond the lamina cribrosa but not to the surgical resection line or exhibits massive choroidal invasion
pT3b	Tumour invades the optic nerve beyond the lamina cribrosa but not to the surgical resection line and exhibits massive choroidal invasion
pT4a	Tumour invades the optic nerve to the resection line, but no extra-ocular extension is identified
pT4b	Tumour invades the optic nerve to the resection line, and extra-ocular extension is identified
