# Supplementary material for: Association of p53 rs1042522, MDM2 rs2279744, and p21 rs1801270 polymorphisms with retinoblastoma risk and invasion in a Chinese population
Source: Sci Rep. 2015 Aug 20;5:13300. doi: 10.1038/srep13300 (PMC4642541; doi:10.1038/srep13300)
Supplement: Supplementary Information [file srep13300-s1.pdf]

Association of *p53* rs1042522, *MDM2* rs2279744, and *p21* rs1801270 polymorphisms with retinoblastoma risk and invasion in a Chinese population

Rongxin Chen,[1] Shu Liu,[2] Huijing Ye,[1] Jiali Li,[3] Yi Du,[4] Lingyan Chen,[5] Xiaoman Liu,[3] Yungang Ding,[1] Qian Li,[1] Yuxiang Mao,[1] Siming Ai,[1] Ping Zhang,[1] Wenfang Ma[1], Huasheng Yang[1*]

[1]State Key Laboratory of Ophthalmology, Zhongshan Ophthalmic Center, Sun Yat-sen University, Guangzhou 510060, China

[2]Sun Yat-sen University Cancer Center; State Key Laboratory of Oncology in South China; Collaborative Innovation Center for Cancer Medicine, Guangzhou 510060, China

[3]Institute of Clinical Pharmacology, School of Pharmaceutical Sciences, Sun Yat-sen University, Guangzhou 510060, China

[4]Department of Ophthalmology, The First Affiliated Hospital of Guangxi Medical University, Nanning, Guangxi 530021, China

[5]Divisions of Genetics and Molecular Medicine, King's College London, Guy's Hospital, London SE1 9RT, UK

***Corresponding author:** Huasheng Yang, MD, PhD, State Key Laboratory of Ophthalmology, Zhongshan Ophthalmic Center, Sun Yat-sen University, Guangzhou 510060, China. E-mail: yanghs64@126.com; Tel.: +8620-87331539; Fax: +8620-87333271.

Supplementary Table S1. Association between the clinical characteristics of patients with retinoblastoma and rs1042522, rs2279744, and rs1801270 in *p53* pathway genes

Variable	Total	*p53* rs1042522 genotypes				*MDM2* rs2279744 genotypes				*p21* rs1801270 genotypes			
		GG	GC	CC	P^a	TT	TG	GG	P^a	CC	CA	AA	P^a
**Gender**													
Male	102	32 (31.4)	51 (50.0)	19 (18.6)	0.776	20 (19.6)	42 (41.2)	40 (39.2)	0.370	26 (25.5)	52 (51.0)	24 (23.5)	0.226
Female	66	21 (31.8)	30 (45.5)	15 (22.7)		14 (21.2)	33 (50.0)	19 (28.8)		25 (37.9)	29 (43.9)	12 (18.2)	
**Age**													
≤ 12 months	64	17 (26.6)	36 (56.2)	11 (17.2)	0.263	13 (20.3)	26 (40.6)	25 (39.1)	0.660	19 (29.7)	32 (50.0)	13 (20.3)	0.931
> 12 months	104	36 (34.6)	45 (43.3)	23 (22.1)		21 (20.2)	49 (47.1)	34 (32.7)		32 (30.8)	49 (47.1)	23 (22.1)	
**Laterality**													
Unilateral	97	36 (37.1)	35 (36.1)	26 (26.8)	**0.001**	25 (25.8)	40 (41.2)	32 (33.0)	0.113	32 (33.0)	42 (43.3)	23 (23.7)	0.328
Bilateral	71	17 (23.9)	46 (64.8)	8 (11.3)		9 (12.7)	35 (49.3)	27 (38.0)		19 (26.8)	39 (54.9)	13 (18.3)	
**Clinical predictors**													
Absent	105	36 (34.3)	47 (44.8)	22 (21.0)	0.490	23 (21.9)	42 (40.0)	40 (38.1)	0.295	34 (32.4)	47 (44.8)	24 (22.9)	0.513
Present	63	17 (27.0)	34 (54.0)	12 (19.0)		11 (17.5)	33 (52.4)	19 (30.2)		17 (27.0)	34 (54.0)	12 (19.0)	
**Lag time**													
≤ 1 month	83	30 (36.1)	36 (43.4)	17 (20.5)	0.706	13 (15.7)	39 (47.0)	31 (37.3)	**0.007**	23 (27.7)	44 (53.0)	16 (19.3)	0.790
> 1 month	45	13 (28.9)	22 (48.9)	10 (22.2)		18 (40.0)	17 (37.8)	10 (22.2)		14 (31.1)	21 (46.7)	10 (22.2)	
**Pre-enucleation treatment**													
No prior	96	37 (38.5)	36 (37.5)	23 (24.0)	**0.013**	22 (22.9)	42 (43.8)	32 (33.3)	0.866	30 (31.2)	44 (45.8)	22 (22.9)	0.328

treatment													
Systemic chemotherapy and/or focal treatment[b]	35	6 (17.1)	23 (65.7)	6 (17.1)		9 (25.7)	16 (45.7)	10 (28.6)		9 (25.7)	21 (60.0)	5 (14.3)	

[a]Chi-square test for the distributions of the genotypes according to the clinical characteristics.

[b]Focal treatment included periocular chemotherapy, laser photocoagulation, cryotherapy, or intra-arterial chemotherapy.

Supplementary Table S2. Multivariate analyses of the association between moderate or high retinoblastoma invasion and the selected SNPs of *p53* pathway genes

Variable	No. of cases[*]	No. of events (%)[*]	Adjusted HR (95% CI)[a*]	*P*[*]	No. of cases[#]	No. of events (%)[#]	Adjusted HR (95% CI)[a#]	*P*[#]
***p53* rs1042522**								
GG/CC	47	31 (66.0)	1.00 (reference)		41	25 (61.0)	1.00 (reference)	
GC	39	18 (46.2)	0.49 (0.25-0.95)	**0.035**	41	20 (48.8)	0.33 (0.16-0.69)	**0.003**
***MDM2* rs2279744**								
TT	21	12 (57.1)	1.00 (reference)		19	10 (52.6)	1.00 (reference)	
TG	34	21 (61.8)	0.98 (0.45-2.11)	0.957	37	24 (64.9)	1.27 (0.53-3.05)	0.586
GG	31	16 (51.6)	1.14 (0.51, 2.56)	0.747	26	11 (42.3)	0.94 (0.36-2.48)	0.902
***p21* rs1801270**								
CC	22	13 (59.1)	1.00 (reference)		26	17 (65.4)	1.00 (reference)	
CA	42	23 (54.8)	1.35 (0.64-2.86)	0.429	42	23 (54.8)	0.97 (0.48-1.95)	0.934
AA	22	13 (59.1)	1.09 (0.48-2.47)	0.831	14	5 (35.7)	0.27 (0.08-0.88)	0.030

[a]Adjusted for age (≤ 12 or > 12 months), gender (male or female), laterality (unilateral or bilateral), clinical predictors (whether presented), ICRB classification (group D or E), lag time (≤ 1 or > 1 months) and pre-enucleation treatment (whether administered).

[*]Analyses for the event of moderate invasion; [#]analyses for the event of high invasion.

Supplementary Table S3. The entire list of subgroup analyses of the association between the selected SNPs of *p53* pathway genes and event-free survival for retinoblastoma invasion

Genotype	Subgroup	Log-rank analysis[a]		Cox regression model[b]	
		Time to RB invasion (median ± SE, months)	*P*	HR (95% CI)	*P*
***p53* rs1042522**					
GG/CC	Lag time ≤ 1 month	0.5 ± 0.1		1.00 (reference)	
GC	Lag time ≤ 1 month	5.6 ± 2.4	0.007	0.64 (0.35-1.15)	0.134
GG/CC	Lag time > 1 month	5.0 ± 1.0		1.00 (reference)	
GC	Lag time > 1 month	8.0 ± 3.5	0.008	0.31 (0.13-0.73)	0.007
GG/CC	No prior treatment	0.5 ± 0.2		1.00 (reference)	
GC	No prior treatment	3.0 ± 0.8	0.004	0.50 (0.30, 0.85)	0.010
GG/CC	Pre-enucleation treatment	13.0 ± 1.0		1.00 (reference)	
GC	Pre-enucleation treatment	18.5 ± 6.0	0.587	0.25 (0.05, 1.14)	0.072
***MDM2* rs2279744**					
TT	Lag time ≤ 1 month	0.8 ± 0.2	reference	1.00 (reference)	
TG	Lag time ≤ 1 month	0.5 ± 0.2	0.514	1.26 (0.61-2.60)	0.529
GG	Lag time ≤ 1 month	1.0 ± 0.2	0.273	1.06 (0.49-2.32)	0.885
TT	Lag time > 1 month	7.0 ± 1.0	reference	1.00 (reference)	
TG	Lag time > 1 month	6.0 ± 0.5	0.270	1.02 (0.41-2.53)	0.970
GG	Lag time > 1 month	4.0 ± 0.8	0.081	1.04 (0.35-3.09)	0.939
TT	No prior treatment	2.0 ± 0.5	reference	1.00 (reference)	
TG	No prior treatment	1.0 ± 0.2	0.582	0.99 (0.54-1.81)	0.970

GG	No prior treatment	1.0 ± 0.3	0.520	0.96 (0.51-1.81)	0.901
TT	Pre-enucleation treatment	26.5 ± 8.6	reference	1.00 (reference)	
TG	Pre-enucleation treatment	13.0 ± 3.2	0.116	1.93 (0.39-9.60)	0.421
GG	Pre-enucleation treatment	11.0 ± 7.2	0.333	1.72 (0.19-15.58)	0.628
***p21* rs1801270**					
CC	Lag time ≤ 1 month	0.5 ± 0.2	reference	1.00 (reference)	
CA	Lag time ≤ 1 month	1.0 ± 0.3	0.076	0.76 (0.41-1.43)	0.402
AA	Lag time ≤ 1 month	0.8 ± 0.2	0.469	0.54 (0.25-1.16)	0.113
CC	Lag time > 1 month	7.0 ± 1.5	reference	1.00 (reference)	
CA	Lag time > 1 month	4.0 ±1.1	0.150	2.27 (1.00-5.15)	0.050
AA	Lag time > 1 month	7.0 ± 1.5	0.745	1.19 (0.42-3.37)	0.743
CC	No prior treatment	1.0 ± 0.3	reference	1.00 (reference)	
CA	No prior treatment	1.0 ± 0.5	0.415	1.30 (0.77-2.21)	0.332
AA	No prior treatment	1.0 ± 0.2	0.791	0.75 (0.38-1.46)	0.397
CC	Pre-enucleation treatment	13.0 ± 2.2	reference	1.00 (reference)	
CA	Pre-enucleation treatment	18.5 ± 5.9	0.711	0.38 (0.07-1.95)	0.245
AA	Pre-enucleation treatment	22.0 ± 4.8	0.428	1.68 (0.23-12.32)	0.610

[a]Crude analysis.

[b]The HRs and *P* values were adjusted for age, gender, laterality, ICRB, clinical predictors, lag time and pre-enucleation treatment.

Abbreviations: SNPs, single nucleotide polymorphisms; SE, standard error.
